# Knee Arthroscopic Surgery in Middle-Aged Patients With Meniscal Symptoms: A 10-Year Follow-up of a Prospective, Randomized Controlled Trial

**DOI:** 10.1177/03635465241255653

**Published:** 2024-08-05

**Authors:** Sofi Sonesson, Ingo Springer, Jafar Yakob, Henrik Hedevik, Håkan Gauffin, Joanna Kvist

**Affiliations:** † Unit of Physiotherapy, Department of Medical and Health Sciences, Linköping University, Linköping, Sweden; ‡ Department of Orthopedics, and Department of Clinical and Experimental Medicine, Linköping University, Linköping, Sweden; § Department of Radiology, and Department of Medical and Health Sciences, Linköping University, Linköping, Sweden; Investigation performed at Linköping University, Linköping, Sweden

**Keywords:** knee arthroscopy, meniscectomy, menisci, middle-aged, radiographic osteoarthritis

## Abstract

**Background:**

Short- and midterm evaluations of arthroscopic meniscal surgery have shown little or no effect in favor of surgery, although long-term effects, including radiographic changes, are unknown.

**Purpose:**

To compare the 10-year outcomes in middle-aged patients with meniscal symptoms between a group that received an exercise program alone and a group that received knee arthroscopy in addition to the exercise program with respect to the prevalence of radiographic and symptomatic osteoarthritis (OA), patient-reported outcomes, and clinical status.

**Study Design:**

Randomized controlled trial; Level of evidence, 1.

**Methods:**

Of 179 eligible patients aged 45 to 64 years, 150 were randomized to undergo either 3 months of exercise therapy (nonsurgery group) or knee arthroscopy in addition to the exercise therapy (surgery group). Surgery usually consisted of partial meniscectomy (n = 56) or diagnostic arthroscopy (n = 8). Radiographs were assessed according to the Kellgren-Lawrence score at the baseline and 5- and 10-year follow-ups. Patient-reported outcome measures were reported at the baseline and 1-, 3-, 5-, and 10-year follow-ups. Clinical status was assessed at a 10-year follow-up. The primary outcomes were radiographic OA and changes in the Knee injury and Osteoarthritis Outcome Score Pain subscale (KOOS_PAIN_) from the baseline to the 10-year follow-up. The primary analysis was performed using the intention-to-treat approach.

**Results:**

At the time of the 10-year follow-up, eight patients had died, leaving 142 eligible patients. Radiographic OA was assessed for 95 patients (67%), questionnaires were answered by 110 (77%), and the clinical status was evaluated for 95 (67%). Radiographic OA was present in 67% of the patients in each group (*P*≥ .999); symptomatic OA was present in 47% of the nonsurgery group and 57% of the surgery group (*P* = .301). There were no differences between groups regarding changes from baseline to 10 years in any of the KOOS subscales.

**Conclusion:**

Knee arthroscopic surgery, in most cases consisting of partial meniscectomy or diagnostic arthroscopy, in addition to exercise therapy in middle-aged patients with meniscal symptoms, did not increase the rates of radiographic or symptomatic OA and resulted in similar patient-reported outcomes at the 10-year follow-up compared with exercise therapy alone. Considering the short-term benefit and no long-term harm from knee arthroscopic surgery, the treatment may be recommended when first-line treatment—including exercise therapy for ≥3 months—does not relieve patient’s symptoms.

**Registration:**

Clinical Trials NCT01288768 (ClinicalTrials.gov identifier).

The use of arthroscopic surgery in the treatment of degenerative meniscal tears is controversial.^3,30^ Systematic reviews with meta-analyses have found contradictory results with evidence of little or no clinically significant benefit from arthroscopic surgery.^25^ One recent review found no difference between patient groups treated with knee arthroscopic surgery or physical therapy,^23^ whereas other reviews^7,21,32,34^ found a small positive effect on pain up to 6 months, 1 year,^1,20^ or 2 years later.^6^ In addition, a recent 5-year follow-up of a randomized controlled trial (RCT), which was not included in the systematic reviews, showed a statistically significant, but not clinically significant, better patient-reported knee function for patients treated with arthroscopic surgery.^24^ A recently published systematic review with meta-analyses utilizing pooled individual participant data from 4 studies showed less pain after knee arthroscopic surgery at a 2-year follow-up.^37^ The current recommendation for treating patients with knee pain related to degenerative meniscal tears is to start with ≥3 months of nonsurgical treatment, such as rehabilitation protocols, before proceeding with arthroscopic partial meniscectomy if this treatment fails.^4^

One consideration opposing surgical treatment is the possibly heightened risk of developing knee osteoarthritis (OA) after the removal of meniscal tissue.^11^ Data from a register-based study shows that joint space narrowing was 25 times greater 1 year after a partial meniscectomy compared with nonsurgical treatment, although no differences were observed during a 6-year follow-up.^27^ The as-treated (AT) analyses of an RCT showed that the risk of total knee replacement (TKR) was 4.9 times higher during the 5-year follow-up in the group receiving arthroscopic partial meniscectomy compared with the physical therapy group.^17^ Using structural cartilage changes detected by magnetic resonance imaging (MRI) as an outcome, Collins et al^8^ found an increased risk of having a worsening score for the cartilage surface in all knee subregions, a worsening effusion-synovitis score, and ≥1 additional subregions with osteophytes after 18 months in the arthroscopic partial meniscectomy group compared with the physical therapy group. At the 5-year follow-up, the only difference between the groups was the worsening of the osteophyte score. Radiographic assessment is considered the gold standard for assessing OA, and long-term radiographic results from RCTs are sparse. Four previous RCTs found no difference in radiographic OA (ROA) between arthroscopic partial meniscectomy and physical therapy at 2- or 5-year^5,15,31,38^ follow-ups, while 1 RCT found a slightly increased risk of ROA progression after arthroscopic partial meniscectomy compared with sham surgery.^29^

The present RCT, which successfully recruited almost all eligible patients in the catchment area,^22^ showed a statistically and clinically significant positive effect on knee pain after knee arthroscopic surgery and exercise therapy at a 1-year follow-up compared with exercise therapy alone.^13^ During the 5-year follow-up, there were no group differences in knee pain or radiographic deterioration.^31^ In this long-term follow-up, the objectives were to compare the 10-year outcomes in middle-aged patients with meniscal symptoms between a group that received an exercise program alone and a group that received knee arthroscopic surgery in addition to the exercise program regarding (1) the prevalence of ROA and symptomatic OA (SOA), (2) the change in patient-reported outcomes (PROs) between the baseline and 10-year follow-ups, and (3) patients’ clinical status. We hypothesized that the surgery group would have a higher prevalence of ROA and SOA than the nonsurgery group and that there would be no differences between the groups relative to changes in PROs at the 10-year follow-up.

## Methods

### Study Design and Participants

**Figure 1.**
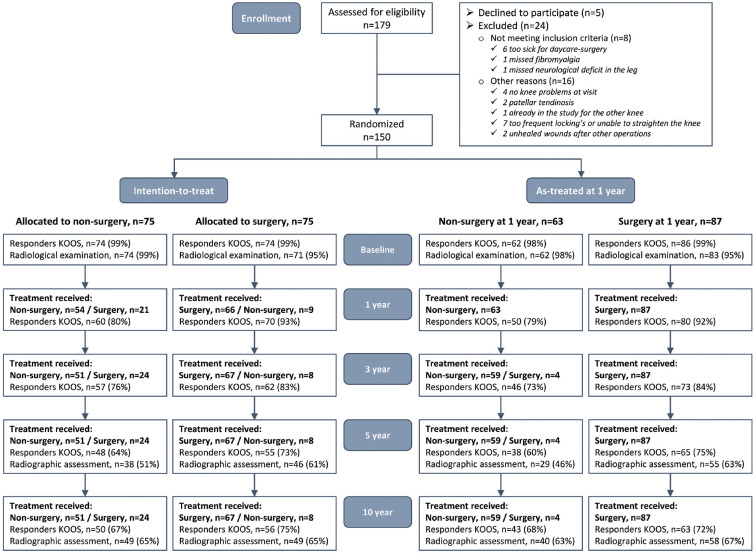
Flowchart of patient enrollment and randomization. The 10-year follow-up data were analyzed using the intention-to-treat and as-treated approaches. For the as-treated analyses, patients who underwent knee arthroscopic surgery in the index knee between their allocation and the 1-year follow-up were included in the surgery group. Patients who did not undergo knee arthroscopic surgery in the index knee during the first year after inclusion were included in the nonsurgery group. KOOS, Knee injury and Osteoarthritis Outcome Score.

Participants were recruited from the orthopaedic department at the Linköping University Hospital between 2010 and 2012. The clinical routine was that middle-aged patients with pain from the knee joint, where the general practitioner suspected a meniscal injury, received standing radiographs and exercise therapy for ≥3 months before they were referred to the orthopaedic department. Eligibility was determined by 1 orthopaedic surgeon (H.G.) who evaluated all referred patients with a suspected meniscal injury. The inclusion criteria were as follows: age 45 to 64 years; symptoms for >3 months; standing radiograph with an Ahlbäck grade of 0 (<50% reduction of the joint space, without consideration of possible osteophytes),^2^ and having undergone ≥3 months of exercise therapy before inclusion. The exclusion criteria were as follows: locked knee or joint locking for >2 seconds more than once a week; rheumatic or neurological diseases; fibromyalgia; hip or knee joint replacements; or a contraindication for day surgery at the current unit (body mass index >35 or a serious medical illness). All consecutive patients who met the inclusion criteria and did not meet any exclusion criteria were invited to participate in the study (Figure 1). Details concerning the recruitment have been published previously.^13^ The study was approved by the Swedish Ethical Review Authority (Dnr: 2010/6-31 and 2020-04157).

The patients were randomly allocated to 1 of 2 parallel intervention groups. Their allocations were placed in sequentially numbered, opaque, sealed envelopes and divided into 15 blocks of 10. After enrollment, the patient and a nurse opened the envelope. The orthopaedic surgeon who enrolled and assessed the patients (H.G.) was blinded to the allocation sequence. The 2 intervention groups were as follows:

1. Nonsurgery: The patients received a physical therapy appointment within 2 weeks, with a functional assessment and instructions for an exercise program. At an independent clinic, 5 physical therapists (S.S.) experienced in knee rehabilitation gave individual instructions for the exercise program. The exercise program aimed to increase muscle function and postural control.^13^
2. Surgery: The patients received the same physical therapy appointment with instructions for exercise therapy within 2 weeks, plus knee arthroscopic surgery within 4 weeks of inclusion. Any significant meniscal injuries were resected during the arthroscopy.

For the 10-year follow-up, patients were invited 10 years after their inclusion in the study to complete a questionnaire, undergo a radiologic examination of both knees, and have an appointment with an orthopaedic surgeon (H.G. and I.S.) for a clinical assessment of knee function. A letter was sent to each patient regarding the follow-up procedure, along with an informed consent form, the questionnaire, and a prepaid return envelope. Up to 3 reminders were sent. Patients could provide informed consent to participate in ≥1 of the 3 study components—questionnaires, radiologic examination, and clinical assessment.

### Interventions

The 3-month exercise intervention took place at an independent physical therapy clinic. The exercise aimed to increase muscle function and postural control. Surgery was performed by 1 of 2 experienced arthroscopists at an independent day surgery clinic. During the arthroscopy, after the arthroscope was inserted in the joint and the joint was visually inspected, the surgeon judged, according to experience, whether a partial meniscal resection or any other surgical treatment was indicated. All patients were allowed to perform full weightbearing activities immediately after surgery.

Of the 75 patients who initially were randomized to surgery, 66 had the index surgery within 1 year—56 patients had partial meniscal resection, there were also 2 removal of degenerated joint cartilage fragments, 1 resection of loose bodies, 1 synovectomy, and 1 partial resection of anterior cruciate ligament remnants, and 8 patients were judged not to need a surgical treatment other than the diagnostic arthroscopy. A punch but not a shaver was used as a standard at meniscal resection. Detailed information regarding the interventions was previously published.^13^

### Outcome Measures

#### Radiographic Assessment

Weightbearing radiographs were performed at baseline and during the 5- and 10-year follow-ups. One radiologist (J.Y.), blinded to the allocation and treatments, assessed all radiographs according to the Kellgren-Lawrence score at the baseline and the 5- and 10-year follow-ups.^18^ The grades were as follows: grade 1, possible osteophytes only; grade 2, definite osteophytes and possible joint space narrowing; grade 3, moderate osteophytes and definite narrowing; and grade 4, large osteophytes, severe joint space narrowing, and/or bony sclerosis. ROA was defined^28^ as grade ≥2. Knee replacement was scored as end-stage knee OA. Progression of knee OA was defined as having ≥1-step deterioration compared with the baseline measurement and having a Kellgren- Lawrence grade of ≥2. SOA was defined as the presence of ROA in the tibiofemoral joint together with the presence of knee pain and/or symptoms defined as a ≥1-step reduction from the maximal score (no knee symptoms) to ≥50% of items within the Knee injury and Osteoarthritis Outcome Score (KOOS) Pain and/or Symptoms subscales.^19^

#### Patient-Reported Outcome Measures

Before randomization (baseline), the orthopaedic surgeon assessed the patient based on the inclusion and exclusion criteria. Then, the patient was isolated to complete the patient-reported outcome measures (PROMs) forms, including the KOOS, the EuroQol 5 dimensions quality of life assessment, and the Physical Activity Scale. Patients completed the same questionnaires at 3 months and 1, 3, 5, and 10 years after baseline. All follow-up questionnaires were completed by the patients at home and returned in an envelope with prepaid postage. There were no planned follow-up visits for the patients at the orthopaedic clinic before the 10-year follow-up.

#### Clinical Status

At the 10-year follow-up visit, 1 of 2 orthopaedic surgeons (H.G. and I.S.) assessed the knee joint status and functional performance. Passive knee range of motion was evaluated using a plastic handheld goniometer. Knee joint effusion and joint tenderness (medial and lateral) were assessed by palpation (yes/no). Symmetric load distribution during gait was visually evaluated and graded as normal gait, slight deterioration, or significant deterioration. Functional performance was assessed with the 30-second chair stand test, during which the patient was asked to complete a maximal number of stands in 30 seconds while fully sitting between each stand. The test was performed on 2 legs and reported as a maximal number, while a test on 1 leg (index leg) was reported as a dichotomous variable, with the patient managing ≥1 repetition or not being able to do so.

### Statistical Analysis

The 10-year follow-up data were analyzed using intention-to-treat (ITT) and AT approaches. For the AT analyses, patients who underwent knee arthroscopic surgery in the index knee between their allocation and the 1-year follow-up were included in the surgery group. Patients who did not undergo knee arthroscopic surgery in the index knee during the first year after inclusion were included in the nonsurgery group (Figure 1).

The primary outcomes were the prevalence of ROA and the change in the KOOS Pain (KOOS_PAIN_) subscale between the baseline and the 10-year follow-up.

Between-group comparisons in patient characteristics— collected at the baseline and the 10-year follow-up—were performed using the independent samples *t* test for normally distributed continuous variables (age) and the Pearson chi-square test for categorical variables (Table 1).

**Table 1:** Patient Characteristics at Baseline and 10-Year Follow-up^*a*^

	Intention-to-Treat			As-Treated at 1 Year		
	Nonsurgery	Surgery	*P*	Nonsurgery	Surgery	*P*
Baseline	n = 75	n = 75		n = 63	n = 87	
Age, y	54.2 (5.9)	54.5 (5.2)	.725^*b*^	54.3 (5.8)	54.4 (5.3)	.929^*b*^
Male sex	42 (56)	40 (53)	.743^*c*^	38 (60)	44 (51)	.237^*c*^
EQ-5D index	0.62 (0.25)	0.63 (0.22)	.913^*b*^	0.66 (0.23)	0.60 (0.24)	.154^*b*^
EQ-5D VAS	63.8 (22.2)	62.1 (19.2)	.631^*b*^	66.6 (21.3)	60.4 (20)	.078^*b*^
Moderate to high physical activity, PAS 4-6	23 (32)^4^	23 (32)^3^	.954^*c*^	19 (32)^4^	27 (32)^3^	.994^*c*^
Total No. of surgeries to index knee			N/A			N/A
0	50 (67)	7 (9)		57 (90)	0	
1	22 (29)	61 (81)		6 (10)	77 (89)	
2	3 (4)	7^*d*^ (9)		0	10^*d*^ (11)	
Total No. of surgeries to the contralateral knee			.261^*c*^			.242^*c*^
0	69 (92)	64 (85)		59 (94)	74 (85)	
1	6^*e*^ (8)	9 (12)		4^*e*^ (6)	11 (13)	
2	0	2 (3)		0	2 (2)	
At a 10-year follow-up	n = 53	n = 57		n = 45	n = 65	
EQ-5D index	0.81 (0.12)	0.75 (0.23)	.097^*b*^	0.79 (0.15)	0.77 (0.21)	.543^*b*^
EQ-5D VAS	76.5 (17.7)	70.6 (18.5)	.094^*b*^	74.8 (18.5)	72.4 (18.2)	.517^*b*^
Moderate to high physical activity (PAS 4-6) during the past month	26 (51)^2^	18 (32)^1^	**.048** ^*c*^	17 (40)^2^	27 (42)^1^	.785^*c*^
BMI, kg/m^2^	26.9 (4.2)	26.2 (4)	.418^*b*^	26.9 (4.3)	26.3 (3.9)	.403^*b*^
Education, university level	24 (46)^1^	21 (38)^2^	.404^*c*^	21 (48)^1^	24 (38)^2^	.321^*c*^
Employment			.242^*c*^			.642^*c*^
Employed/job seeker	30 (58)^1^	26 (46)^1^		24 (55)^1^	32 (50)^1^	
Retired/other	22 (42)	30 (54)		20 (45)	32 (50)	
Employment, load			.378^*c*^			.693^*c*^
Light work	12 (23)^1^	10 (18)^1^		17 (39)^1^	22 (34)^1^	
Mobile work	10 (19)	7 (13)		14 (32)	18 (28)	
Heavy work	15 (29)	17 (30)		13 (30)	24 (38)	
Current or former smoker	20 (39)^1^	26 (48)^3^	.314^*c*^	17 (39)^1^	29 (47)^3^	.405^*c*^
High blood pressure	19 (37)^1^	20 (36)^1^	.929^*c*^	15 (34)^1^	24 (38)^1^	.717^*c*^
Diabetes	5 (10)^1^	4 (7)^1^	.642^*c*^	3 (7)^1^	6 (9)^1^	.637^*c*^
Cardiovascular disease	4 (8)^1^	3 (5)^1^	.622^*c*^	3 (7)^1^	4 (6)^1^	.906^*c*^
Neurological disease	1 (2)^1^	5 (9)^1^	.112^*c*^	1 (2)^1^	5 (8)^1^	.217^*c*^
Cancer	3 (6)^1^	3 (5)^1^	.926^*c*^	1 (2)^1^	5 (8)^1^	.217^*c*^
Dementia	0^1^	1 (2)^1^	.333^*c*^	0^1^	1 (2)^1^	.405^*c*^

a Data are presented as mean (SD) or n (valid %). Bold value indicates *P* < .05. Superscript numbers indicate the number of patients with missing values. BMI, body mass index; EQ-5D, EuroQol 5 dimensions; N/A, not applicable; PAS, Physical Activity Scale; TKR, total knee replacement; VAS, visual analog scale.

b Independent-samples *t* test.

c Pearson chi-square test.

d 3 TKRs.

e 1 TKR.

Between-group comparisons in the prevalence of ROA and SOA in both the index knee and the contralateral knee were performed using the Pearson chi-square test or the Fisher exact test at the baseline and at 5- and 10-year follow-ups (Table 2).

**Table 2:** Knee Osteoarthritis at Baseline, 5- and 10-Year Follow-up Based on the 10-Year Follow-up Cohort^*a*^

		Index Knee									Contralateral Knee		
		Baseline			5 Years			10 Years			10 Years		
		Nonsurgery	Surgery	*P*	Nonsurgery	Surgery	*P*	Nonsurgery	Surgery	*P*	Nonsurgery	Surgery	*P*
Intention-to-Treat	Cohort	n = 48	n = 46		n = 29	n = 34		n = 49	n = 49		n = 47	n = 49	
	K-L			.104^*b*^			.106^*c*^			.412^*c*^			.933^*b*^
	Grade 0	11 (23)	20 (43)		1 (3)	6 (18)		3 (6)	2 (4)		5 (11)	6 (12)	
	Grade 1	12 (25)	9 (20)		2 (7)	3 (9)		13 (27)	14 (30)		21 (46)	18 (37)	
	Grade 2	25 (52)	17 (37)		12 (41)	15 (44)		13 (27)	12 (26)		9 (20)	11 (22)	
	Grade 3				14 (48)	8 (24)		16 (33)	9 (20)		6 (13)	7 (14)	
	Grade 4				0	2 (6)		4 (8)	9 (20)		5 (11)	7 (14)	
	Knee replacement				0	0		0	3		1	0	
	ROA, K-L ≥2	25 (52)	17 (37)	.140^*b*^	26 (90)	25 (74)	.104^*b*^	33 (67)	33 (67)	≥.999^*b*^	21 (45)	25 (51)	.534^*c*^
	SOA	25 (52)	17 (37)	.140^*b*^	10 (36)^1^	18 (55)^1^	.141^*b*^	21 (47)^2^	27 (57)^4^	.301^*b*^	12 (27)	21 (44)	.100^*b*^
	Progression	N/A	N/A		15 (52)	17 (50)	.891^*b*^	23 (47)	29 (59)	.225^*b*^	N/A	N/A	
As-Treated at 1 Year	Cohort	n = 40	n = 54		n = 22	n = 41		n = 40	n = 58		n = 39	n = 57	
	K-L			**.032** ^*b*^			.473^*c*^			.675^*c*^			.629^*c*^
	Grade 0	8 (20)	23 (43)		1 (5)	6 (15)		2 (5)	3 (5)		3 (8)	8 (14)	
	Grade 1	13 (33)	8 (15)		2 (9)	3 (7)		12 (30)	15 (27)		19 (50)	20 (35)	
	Grade 2	19 (48)	23 (43)		12 (55)	15 (37)		12 (30)	13 (24)		8 (21)	12 (21)	
	Grade 3				7 (32)	15 (37)		11 (28)	14 (25)		4 (11)	9 (16)	
	Grade 4				0	2 (5)		3 (8)	10 (18)		4 (11)	8 (14)	
	Knee replacement				0	0		0	3		1	0	
	ROA, K-L ≥2	19 (48)	23 (43)	.636^*b*^	19 (86)	32 (78)	.516^*c*^	26 (65)	40 (69)	.681^*b*^	17 (44)	29 (51)	.493^*b*^
	SOA	19 (48)	23 (43)	.636^*b*^	8 (38)^1^	20 (50)^1^	.375^*b*^	17 (46)^3^	31 (56)^3^	.327^*b*^	11 (30)^2^	22 (40)^2^	.314^*b*^
	Progression	N/A	N/A		9 (41)	23 (56)	.250^*b*^	17 (43)	35 (60)	.082^*b*^	N/A	N/A	

a Data are presented as mean (valid %). Bold value indicates *P* < .05. Superscript numbers indicate the number of patients with missing values. Cohort, the number of participants who underwent radiological examination or had knee replacement; K-L, Kellgren-Lawrence grade; N/A, not applicable; ROA, radiographic osteoarthritis defined as having ≥2 grade in K-L, including patients with knee replacement; SOA, symptomatic knee osteoarthritis defined as the presence of ROA in the tibiofemoral joint together with the presence of knee pain and/or symptoms defined as a ≥1-step decrease from the best response to ≥50% of items within the Knee injury and Osteoarthritis Outcome Score Pain and/or Symptoms subscales. Progression of knee osteoarthrisis was defined as having ≥2 grade in K-L and ≥1-step deterioration compared with the baseline measurement (patients with knee replacement were not included).

b Pearson chi-square test.

c The Fisher exact test.

Because of the missing data in the KOOS subscales at the various follow-ups, we conducted missing pattern analyses on the main outcome KOOS_PAIN_, comparing completers and noncompleters (participants with missing data in ≥1 follow-up) both within and between the nonsurgery and surgery groups using the independent-samples *t* test. In addition, between-group comparisons of the proportion of completes in the KOOS_PAIN_ were analyzed using the Pearson chi-square test.

Linear mixed models were used to analyze repeated measures of KOOS subscales with time points (baseline, 1-, 3-, 5-, and 10-year follow-ups) and intervention groups (nonsurgery, surgery) treated as fixed effects. The restricted maximum likelihood estimate was used in the linear mixed models, allowing all participants with ≥1 observation to be included in the models under the assumption of data missing at random. The unstructured covariance structure was applied in all models to measure the association among the repeated measures. Bonferroni correction was used on all multiple pairwise contrasts between the 5 time points (see Appendices 1 and 2, available in the online version of this article).

A priori sample size calculation was performed before the commencement of the RCT. A minimal clinically important change of 8 to 10 is considered appropriate for the KOOS_PAIN_. A 10-point change was used as the cutoff, indicating improvement.^26^ To detect a between-group difference of 10 points^26^ (SD, 19) in the KOOS_PAIN_ (α = .05; β = 0.8), we included 75 patients in each group; this accounted for a crossover and dropout rate of 33%.

All statistical analyses were performed in IBM SPSS Statistics for Windows Version 29.0. (IBM Corp). A significant level of .05 was used in all analyses.

## Results

### Study Participants

Patients were randomly assigned to either the surgery (n = 75) or nonsurgery (n = 75) group. At 1 year, 9 patients assigned to surgery crossed over to the nonsurgery group, and 19 patients assigned to nonsurgery underwent knee surgery during the first year and thus crossed over to the surgery group. Within 10 years from the baseline, 68 (91%) of the 75 patients assigned to the surgery group and 25 (33%) of the patients assigned to the nonsurgery group underwent arthroscopy to the index knee, of whom 7 patients underwent 1 additional knee arthroscopy. Three patients had TKR in the index knee, 14 (9%) patients underwent arthroscopy to the contralateral knee, and 1 patient had TKR (Tables 1 and 2, Figure 1).

At the 10-year follow-up, 8 patients had died, leaving 142 eligible patients. A total of 95 patients (67%) underwent weightbearing radiographs and 3 patients had TKR, 110 patients (77%) completed the follow-up questionnaires, and 95 (67%) underwent the functional assessment (Figure 1).

### Patient Characteristics

Patient characteristics at baseline and at the 10-year follow-up are presented in Table 1. At the 10-year follow-up, nearly half of the patients had retired. More patients in the nonsurgical group (ITT analysis) were more physically active at the 10-year follow-up (26; 51%) compared with the surgical group (18; 32%) (*P* = .048). There were no differences in the physical activity level between groups at baseline. A total of 46 patients (32%) reported that they had used tobacco for ≥6 months at some point in their life.

### Radiographic Findings

Three patients in the surgery group had TKR on the index knee 6 (1 patient) and 10 (2 patients) years after their inclusion in the present study.

*ITT Analyses*. Two out of every 3 patients (67% in each group; *P*≥ .999) had ROA, and approximately half the patients (nonsurgery group, 47%; surgery group, 57%; *P* = .301) had SOA, with no differences in ROA or SOA between the groups (Table 2).

*AT Analyses*. ROA was present in 65% of the patients in the nonsurgery group, and 69% in the surgery group had ROA (*P* = .681). SOA was present in 46% of the patients in the nonsurgery group and 56% in the surgery group (*P* = .327) (Table 2).

### Patient-Reported Outcome Measures

*ITT Analyses*. No differences were found between the groups in any of the PROMs at the baseline. At the 1-year follow-up, the nonsurgery group had improved less in the KOOS_PAIN_ (mean change, 18 points [95% CI, 12 to 25] vs 30 points [95% CI, 23 to 36]; *P* = .002), the KOOS Sport and Recreation subscale (KOOS_SPORT_), and the KOOS Quality of Life (KOOS_QOL_), and they scored worse in the KOOS_PAIN_ (mean change, 77 [95% CI, 73 to 81] vs 84 [95% CI, 80 to 88]; *P* = .011) and the KOOS_QOL_ compared with the surgery group. At the 3-year follow-up, the nonsurgery group had tended to improve less (mean difference, 8 points [95% CI, −16 to 0]; *P* = .053) compared with the surgery group. There was no difference in the KOOS_PAIN_ between the nonsurgery and surgery groups (mean, 78 [95% CI, 73 to 81] vs 83 [95% CI, 78 to 87] P = .242) at the 3-year follow-up.

**Figure 2.**
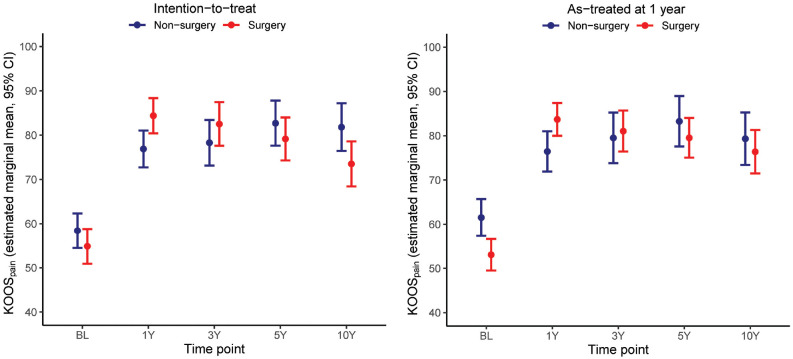
KOOS_PAIN_ scores at baseline, 1-, 3-, 5-, and 10-year follow-ups according to the treatment group in the intention-to-treat and as-treated analyses. KOOS_PAIN_, Knee injury and Osteoarthritis Outcome Score Pain subscore.

At the 10-year follow-up, all patients had improved from baseline (*P* < .05). There were no significant between-group differences regarding changes in scores from baseline to the 10-year follow-up for any of the KOOS subscales. The nonsurgery group scored better in the KOOS_PAIN_ compared with the surgery group (mean, 82 [95% CI, 76 to 87] vs 74 [95% CI, 68 to 79]; *P* = .028) (see Appendices 1 and 2, available online; Figure 2).

*AT Analyses*. At baseline, the nonsurgery group scored better in the KOOS_PAIN_ (mean, 62 [95% CI, 57-66]) vs 53 [95% CI, 49-57]; *P* = .003), the KOOS Activities of Daily Living, the KOOS_SPORT_, and the KOOS_QOL_ (*P* < .05) compared with the surgery group. The nonsurgery group improved less in all KOOS subscales between the baseline and the 1-year follow-up and between the baseline and the 3-year follow-up, except for the KOOS_SPORT_. During the 1-year follow-up, the nonsurgery group scored worse in the KOOS_PAIN_ than the surgery group. All patients had improved from the baseline at the 10-year follow-up (*P* < .05). There were no significant between-group differences in changes in any of the KOOS subscales from the baseline to the 10-year follow-up (see Appendices 1 and 2, available online, and Figure 2).

### Clinical Status

There were no significant differences between the 2 groups in the functional assessments at the 10-year follow-up, except in the single-leg 30-second chair stand test, during which more patients in the nonsurgically treated group in the AT analysis could perform ≥1 repetition (45% vs 20%; *P* = .012) (Table 3).

**Table 3:** Clinical Status of the Injured Knee at the 10-Year Follow-up^*a*^

	Intention-to-Treat			As-Treated at 1 Year		
	Nonsurgery	Surgery	*P*	Nonsurgery	Surgery	*P*
Cohort	n = 48	n = 47		n = 39	n = 56	
Passive ROM						
Flexion	134.3 (7)	132.4 (10.4)^2^	*.305* ^*b*^	134.1 (7)	132.8 (10)^2^	*.464* ^*b*^
Extension	−1.9 (4.1)	−1.8 (3.9)^2^	*.888* ^*b*^	−2.3 (4.5)	−1.5 (3.6)^2^	*.313* ^*b*^
Flexion + extension	132.4 (8.3)	130.6 (12.8)^2^	*.428* ^*b*^	131.8 (9.6)	131.3 (11.6)^2^	*.820* ^*b*^
Knee joint effusion	7 (15)	12 (26)	.182^*c*^	6 (15)	13 (23)	.348^*c*^
Knee joint tenderness						
Medial	11 (23)	15 (32)	.325^*c*^	11 (28)	15 (27)	.879^*c*^
Lateral	5 (10)^1^	6 (13)^1^	.720^*c*^	7 (18)	4 (7)	.105^*c*^
Gait pattern, symmetrical load distribution			.255^*c*^			.255^*c*^
Normal gait	36 (77)	30 (64)		30 (77)	36 (65)	
Slight deviation	10 (21)	13 (28)		7 (18)	16 (29)	
Significant deviation	1 (2)	4 (9)		2 (5)	3 (5)	
30-sec chair stand test, max repetitions						
2 legs	14.1 (5.2)^2^	12.3 (5.8)^1^	.126^*b*^	13.8 (5.6)^1^	12.8 (5.5)^2^	.412^*b*^
Injured leg, >0 repetitions	17 (37)^2^	11 (24)^1^	.174^*c*^	17 (45)^1^	11 (20)^2^	**.012** ^*c*^

a Data are presented as mean (SD) or n (valid %). Bold value indicates *P* < .05. Superscript numbers indicate the number of patients with missing values. ROM, range of motion.

b Independent-samples *t* test.

c Pearson chi-square test.

### Adverse Events

None of the groups reported any adverse events or complications after the arthroscopic surgery or the exercise therapy. However, the results reported that some patients had delayed or had additional knee surgery.

### Response and Dropout Analysis

In the ITT and AT analyses, there were no differences between responders and nonresponders regarding the treatment group (nonsurgery/surgery), sex, age, baseline radiographs, or KOOS at baseline. Nonresponders were less physically active at baseline compared with responders. Also, 17% of the nonresponders engaged in a high level of physical activity (Physical Activity Scale, 4-6) compared with 37% of the responders (*P* = .029).

## Discussion

This 10-year follow-up of the randomized study on exercise therapy alone compared with knee arthroscopic surgery and the same exercise therapy in middle-aged patients with meniscal symptoms showed no differences between treatment groups in ROA or SOA. After 1 year, the patients in the nonsurgery group reported significantly more pain compared with the surgery group.^13^ As during the 5-year follow-up,^31^ no significant differences were found regarding changes in knee pain or overall knee function from the baseline to the 10-year follow-up, although the nonsurgically treated group reported less knee pain (ie, higher KOOS_PAIN_ score) compared with the surgically treated group. In addition, the linear mixed model analyses used in this study showed a clinically relevant benefit obtained from surgery during the 3-year follow-up in the AT analysis.

In our study, patients treated with knee arthroscopic surgery in addition to exercise therapy had no increased risk of ROA 10 years after the treatment compared with patients treated with exercise therapy alone; therefore, our hypothesis that the surgery group would have a higher prevalence of ROA was rejected. This applied to both the ITT and the AT analyses. Our results align with previous^38^ 2- and 5-year^5,15^ follow-ups of RCTs comparing knee arthroscopic surgery with exercise therapy. The argument against knee arthroscopic surgery in this patient group is mainly based on previous evidence of similar PROs from both surgical and nonsurgical treatment^3^ as well as the risk of ROA after knee arthroscopic surgery.^4,27^ However, neither our RCT nor any previous RCT study has shown an increased risk of ROA after knee arthroscopic surgery when compared with exercise therapy. A recent study suggested that patients who underwent arthroscopic partial meniscectomy had a similar risk of knee arthroplasty within a 5-year period compared with those who underwent nonoperative management, except for patients >70 years old.^14^

In our population aged 55 to 75 years at the time of the 10-year follow-up, 67% of the patients in both treatment groups had ROA in the index knee. That is nearly 3 times more prevalent than the general Swedish population, for which ROA prevalence is 17% in the 56- to 64-year age group and 20% in the 65- to 74-year age group. Turkiewicz et al.^15^ showed that approximately 25% of the population had frequent knee pain during the past 12 months; of those, 43% had ROA.^33^ In this study, patients were referred to the orthopaedic clinic 10 years previously because of knee pain still present despite ≥3 months of physical therapy. In addition, during the study period, many patients developed knee problems in their contralateral knee. Fifteen (10%) patients underwent knee surgery on the contralateral knee (including 1 TKR), and 46 (48%) patients had ROA in the contralateral knee. As a result, the higher prevalence of ROA in our selected population compared with the general population is not surprising. Although the definition of early OA is under development, our middle-aged patients with meniscal symptoms included in the study were in the early stages of OA.^3^ The majority progressed to having ROA within 10 years, with no difference between treatment groups.

Both treatment groups reported less pain (higher KOOS_PAIN_ score) during the 10-year follow-up compared with baseline, with no difference in change between the groups (primary outcome). In both the ITT and the AT analyses, knee arthroscopic surgery followed by exercise therapy reduced knee pain and improved overall results, as demonstrated in 4 of the 5 KOOS subscales at 1 year compared with exercise therapy alone. At the 3-year follow-up, there was a tendency toward larger pain reduction for the surgery group in the ITT analyses (8-point difference; *P* = .053) and significantly larger pain reduction in the AT analysis (10-point difference; *P* = .015) compared with the exercise program alone. This is in line with previous studies.^1,6,20^ Our patients who received knee arthroscopic surgery in addition to exercise therapy had similar clinical status compared with patients who received exercise therapy alone and had better results with regard to secondary outcomes such as knee function and quality of life as reported by the other KOOS subscales during the short-term follow-up. The favorable short-term results regarding pain and function after knee arthroscopic surgery may enable participation in physical activity and continued heavy labor, making it worth while undergoing surgery despite the lack of long-term benefits.

One inclusion criterion for this study was that patients had to have undergone ≥3 months of exercise therapy before inclusion. Hence, the patients were initially nonresponders to exercise therapy. Thus, according to present recommendations,^4^ all our patients should have been eligible for knee arthroscopy. Nevertheless, both treatment groups experienced decreased pain levels and improved in all KOOS subscale scores from the baseline through all the follow-ups, meaning that the nonsurgery group who received a new physical therapy period with a structured exercise program also reported less pain. One reason may be insufficient initial exercise therapy before being referred to the orthopaedic department. Another possible explanation may be that being included in a research study made the patient feel better and have better outcomes.^16^ In addition, because the patients had severe knee problems when they sought health care and knee problems can fluctuate over time, an improvement detected during the follow-ups may result from the regression to the mean.^12^

At the 10-year follow-up, the ITT analysis showed that patients in the nonsurgery group had less pain compared with the surgery group (8 points; *P* = .028). There was no difference in the KOOS_PAIN_ in the AT analysis. Ten years after their inclusion in the study, one-third of the patients (n = 25 [33%]) allocated to the nonsurgery group had undergone delayed knee arthroscopy. The 21 (28%) patients who crossed over to have knee arthroscopic surgery during the first year after inclusion had more pain and worse knee function compared with the patients who remained in the nonsurgical group, which is in accordance with a previous study.^37^ Hence, physical therapy alone may be insufficient for some patients with significant pain and meniscal symptoms.

According to the recommendations, the first-line treatment for patients with meniscal symptoms should be exercise therapy.^4^ Although first-line treatment is sufficient for many patients, there are still some patients who benefit from knee arthroscopic surgery. According to the recommendations, it is important to identify and manage knee problems for these patients early to prevent deterioration and the progression of ROA.^36^ Unfortunately, the results from our study cannot point out which treatment is favorable to prevent ROA because two-thirds of the patients in both treatment groups developed ROA. Before inclusion, patients had received exercise therapy in primary care for ≥3 months. Despite our patients being initially nonresponders to physical therapy, they improved after a new, structured session of exercise therapy guided by a physical therapist experienced in knee rehabilitation. This finding highlights that the first-line treatment for these patients needs to be structured and evidence-based. For patients who still do not respond to exercise therapy, knee arthroscopic surgery is a treatment option that does not seem to increase the risk of ROA.

The major strength of our study is that we recruited almost all eligible patients during the study period, with only 5 declining to participate. In addition, the same radiologist, blinded to the treatment received, assessed the radiographs for all the follow-ups.

### Limitations

Approximately one-third of the patients in the nonsurgery group underwent delayed knee arthroscopy, making it difficult to compare the 2 groups. We dealt with this by utilizing both ITT and AT analyses. Regardless, having patients cross over to receive a delayed knee arthroscopic surgery mirrors clinical reality because some patients may not respond to exercise therapy alone. Not all participants had radiographs (67%) or responded to the questionnaires (77%) during the follow-ups. Our sensitivity analyses showed that nonresponders were not significantly different from the responders in baseline characteristics, except for their physical activity level. We analyzed our data with a linear mixed model for the PROs that handled missing data by maximum likelihood estimation.

Although most patients in the surgery group had a partial meniscectomy, the fact that various surgical procedures were performed should be taken into account when interpreting the results. During the arthroscopy, the surgeon inspected the joint and performed a partial meniscal resection or other surgical intervention as indicated. This strategy reflected the clinical routine at the time of recruitment. Surgical interventions were not based on MRI findings because of the high prevalence of incidental meniscal tears in middle-aged and elderly patients^9,10^ and discordant findings between MRI and arthroscopic evaluation of the knee meniscus.^35^

### Future Directions

Two-thirds of the patients in both treatment groups developed ROA. Future research should focus on interventions to prevent knee OA development and progression and improve knee function in this patient group.

## Conclusion

Knee arthroscopic surgery, in most cases consisting of partial meniscectomy or diagnostic arthroscopy, in addition to exercise therapy in middle-aged patients with meniscal symptoms, did not increase the rates of radiographic or symptomatic OA and resulted in similar PROMs at the 10-year follow-up compared with exercise therapy alone. Considering the short-term benefit and no long-term harm from knee arthroscopic surgery, the treatment may be recommended for patients when first-line treatment—including exercise therapy for ≥3 months—does not relieve the patient’s symptoms.

## Supplemental Material

sj-pdf-1-ajs-10.1177_03635465241255653 – Supplemental material for Knee Arthroscopic Surgery in Middle-Aged Patients With Meniscal Symptoms: A 10-Year Follow-up of a Prospective, Randomized Controlled Trial Supplemental material, sj-pdf-1-ajs-10.1177_03635465241255653 for Knee Arthroscopic Surgery in Middle-Aged Patients With Meniscal Symptoms: A 10-Year Follow-up of a Prospective, Randomized Controlled Trial by Sofi Sonesson, Ingo Springer, Jafar Yakob, Henrik Hedevik, Håkan Gauffin and Joanna Kvist in The American Journal of Sports Medicine
