# Supplementary material for: Knee Arthroscopic Surgery in Middle-Aged Patients With Meniscal Symptoms: A 10-Year Follow-up of a Prospective, Randomized Controlled Trial
Source: Am J Sports Med. 2024 Aug 5;52(9):2250–9. doi: 10.1177/03635465241255653 (PMC11308265; doi:10.1177/03635465241255653)
Supplement: sj-pdf-1-ajs-10.1177_03635465241255653 – Supplemental material for Knee Arthroscopic Surgery in Middle-Aged Patients With Meniscal Symptoms: A 10-Year Follow-up of a Prospective, Randomized Controlled Trial [file sj-pdf-1-ajs-10.1177_03635465241255653.pdf]

Appendix 1. Within-group change, and between-group effects of Knee Injury and Osteoarthritis Outcome Score based on linear mixed models (5 time points × 2 groups).

	KOOS	Group	Time points, mean (95% CI); p-value					Within-group change, mean (95% CI); p-value			
			BL	1 y	3 y	5 y	10 y	1 y – BL	3 y – BL	5 y – BL	10 y – BL
Intention-to-treat	**Pain**	Non-surgery	58 (54 to 62)	77 (73 to 81)	78 (73 to 83)	83 (78 to 88)	82 (76 to 87)	18 (12 to 25); p <.001	20 (13 to 27); p <.001	24 (17 to 31); p <.001	23 (15 to 31); p <.001
		Surgery	55 (51 to 59)	84 (80 to 88)	83 (78 to 87)	79 (74 to 84)	74 (68 to 79)	30 (23 to 36); p <.001	28 (21 to 35); p <.001	24 (17 to 31); p <.001	19 (11 to 26); p <.001
		Between-group effects (NS – S)	4 (-2 to 9); p =.210	-7 (-13 to -2); p =.011	-4 (-11 to 3); p =.242	4 (-3 to 11); p =.320	8 (1 to 16); p =.028	-11 (-18 to -4); p =.002	-8 (-16 to 0); p =.053	0 (-8 to 8); p =.999	5 (-4 to 13); p =.276
	**Symptoms**	Non-surgery	62 (58 to 66)	78 (74 to 82)	78 (73 to 83)	80 (75 to 86)	79 (73 to 84)	16 (10 to 21); p <.001	16 (9 to 23); p <.001	19 (12 to 25); p <.001	17 (9 to 24); p <.001
		Surgery	59 (55 to 63)	82 (78 to 86)	82 (77 to 86)	77 (72 to 82)	71 (66 to 77)	23 (18 to 29); p <.001	23 (17 to 30); p <.001	18 (11 to 24); p <.001	12 (5 to 20); p <.001
		Between-group effects (NS – S)	3 (-3 to 9); p =.274	-4 (-10 to 1); p =.134	-4 (-11 to 3); p =.238	4 (-3 to 11); p =.284	8 (0 to 15); p =.059	-8 (-14 to -1); p =.017	-7 (-15 to 0); p =.052	1 (-7 to 8); p =.862	4 (-4 to 12); p =.300
	**Function in daily living**	Non-surgery	68 (64 to 73)	82 (78 to 86)	82 (77 to 87)	86 (81 to 91)	84 (79 to 89)	14 (8 to 20); p <.001	14 (7 to 20); p <.001	18 (11 to 24); p <.001	16 (8 to 23); p <.001
		Surgery	65 (61 to 69)	86 (82 to 90)	86 (82 to 91)	83 (79 to 88)	77 (72 to 82)	21 (15 to 27); p <.001	21 (15 to 27); p <.001	18 (12 to 25); p <.001	12 (5 to 19); p <.001
		Between-group effects (NS – S)	3 (-3 to 9); p =.273	-4 (-10 to 2); p =.168	-4 (-11 to 2); p =.220	3 (-4 to 9); p =.427	7 (0 to 14); p =.054	-8 (-14 to -1); p =.023	-7 (-14 to 0); p =.036	-1 (-8 to 7); p =.827	4 (-5 to 12); p =.374
	**Function in sport and recreation**	Non-surgery	32 (27 to 37)	53 (47 to 60)	59 (52 to 67)	60 (52 to 68)	55 (47 to 63)	21 (12 to 30); p <.001	27 (18 to 37); p <.001	28 (18 to 37); p <.001	23 (12 to 34); p <.001
		Surgery	29 (24 to 35)	59 (53 to 65)	61 (53 to 68)	55 (48 to 62)	46 (39 to 54)	30 (21 to 38); p <.001	31 (22 to 40); p <.001	26 (16 to 35); p <.001	17 (7 to 27); p <.001
		Between-group effects (NS – S)	3 (-5 to 10); p =.450	-6 (-15 to 4); p =.229	-1 (-12 to 9); p =.793	5 (-6 to 15); p =.382	9 (-2 to 20); p =.123	-8 (-18 to 1); p =.077	-4 (-14 to 6); p =.419	2 (-9 to 12); p =.726	6 (-5 to 17); p =.298
	**Knee related Quality of life**	Non-surgery	36 (32 to 39)	58 (52 to 63)	64 (57 to 70)	65 (58 to 71)	64 (58 to 71)	22 (14 to 30); p <.001	28 (20 to 36); p <.001	29 (20 to 38); p <.001	29 (19 to 38); p <.001
		Surgery	34 (30 to 38)	66 (61 to 71)	70 (64 to 76)	65 (59 to 71)	59 (53 to 66)	32 (24 to 40); p <.001	36 (28 to 44); p <.001	31 (23 to 39); p <.001	26 (17 to 34); p <.001
		Between-group effects (NS – S)	2 (-4 to 7); p =.555	-8 (-16 to -1); p =.036	-6 (-15 to 2); p =.149	0 (-9 to 8); p =.922	5 (-5 to 14); p =.304	-10 (-19 to -1); p =.027	-8 (-17 to 1); p =.082	-2 (-11 to 7); p =.663	3 (-7 to 13); p =.517

As-treated	**Pain**	Non-surgery	62 (57 to 66)	76 (72 to 81)	80 (74 to 85)	83 (78 to 89)	79 (73 to 85)	15 (8 to 22); p <.001	18 (10 to 26); p <.001	22 (14 to 30); p <.001	18 (9 to 26); p <.001
		Surgery	53 (49 to 57)	84 (80 to 87)	81 (76 to 86)	80 (75 to 84)	76 (71 to 81)	31 (25 to 36); p <.001	28 (22 to 34); p <.001	26 (20 to 33); p <.001	23 (16 to 31); p <.001
		Between-group effects (NS – S)	8 (3 to 14); p =.003	-7 (-13 to -1); p =.016	-2 (-9 to 6); p =.680	4 (-4 to 11); p =.310	3 (-5 to 11); p =.450	-16 (-23 to -9); p <.001	-10 (-18 to -2); p =.015	-5 (-13 to 3); p =.239	-6 (-14 to 3); p =.214
	**Symptoms**	Non-surgery	63 (59 to 68)	77 (73 to 81)	78 (73 to 83)	81 (76 to 87)	76 (70 to 82)	14 (8 to 20); p <.001	15 (7 to 22); p <.001	18 (11 to 26); p <.001	13 (5 to 21); p <.001
		Surgery	58 (54 to 62)	82 (78 to 85)	81 (77 to 85)	77 (72 to 81)	74 (69 to 79)	24 (19 to 29); p <.001	23 (17 to 29); p <.001	19 (13 to 25); p <.001	16 (9 to 23); p <.001
		Between-group effects (NS – S)	5 (-1 to 11); p =.084	-5 (-10 to 1); p =.098	-3 (-10 to 4); p =.375	5 (-3 to 12); p =.214	2 (-6 to 10); p =.557	-10 (-16 to -4); p =.002	-8 (-16 to -1); p =.031	-1 (-8 to 7); p =.876	-3 (-11 to 5); p =.501
	**Function in daily living**	Non-surgery	72 (67 to 76)	81 (76 to 86)	82 (77 to 87)	86 (81 to 91)	82 (77 to 88)	9 (3 to 15); p =.001	10 (3 to 17); p =.001	14 (6 to 21); p <.001	10 (2 to 19); p =.007
		Surgery	63 (59 to 67)	86 (82 to 90)	85 (81 to 90)	84 (80 to 88)	79 (74 to 84)	23 (18 to 28); p <.001	23 (17 to 28); p <.001	21 (15 to 27); p <.001	16 (9 to 23); p <.001
		Between-group effects (NS – S)	9 (3 to 15); p =.003	-5 (-11 to 1); p =.091	-3 (-10 to 3); p =.323	2 (-5 to 8); p =.579	3 (-4 to 11); p =.400	-14 (-20 to -8); p <.001	-12 (-19 to -5); p <.001	-7 (-15 to 0); p =.055	-6 (-14 to 3); p =.169
	**Function in sport and recreation**	Non-surgery	37 (31 to 42)	55 (48 to 63)	60 (52 to 69)	58 (50 to 67)	56 (47 to 65)	18 (9 to 28); p <.001	24 (13 to 34); p <.001	21 (11 to 32); p <.001	19 (8 to 31); p <.001
		Surgery	26 (22 to 31)	57 (51 to 63)	60 (53 to 66)	57 (50 to 63)	47 (40 to 54)	30 (23 to 38); p <.001	33 (25 to 41); p <.001	30 (22 to 39); p <.001	21 (11 to 30); p <.001
		Between-group effects (NS – S)	10 (3 to 18); p =.005	-2 (-11 to 8); p =.730	1 (-10 to 12); p =.873	2 (-9 to 13); p =.765	9 (-2 to 21); p =.118	-12 (-21 to -3); p =.013	-10 (-20 to 1); p =.072	-9 (-20 to 2); p =.112	-1 (-13 to 10); p =.831
	**Knee related Quality of life**	Non-surgery	39 (35 to 43)	58 (52 to 65)	64 (58 to 71)	65 (58 to 72)	65 (57 to 72)	19 (10 to 28); p <.001	25 (16 to 34); p <.001	25 (16 to 35); p <.001	25 (15 to 36); p <.001
		Surgery	31 (28 to 35)	64 (59 to 69)	68 (63 to 74)	65 (59 to 71)	60 (54 to 66)	33 (26 to 40); p <.001	37 (30 to 44); p <.001	34 (26 to 41); p <.001	28 (20 to 37); p <.001
		Between-group effects (NS – S)	8 (2 to 13); p =.005	-6 (-14 to 2); p =.152	-4 (-13 to 5); p =.377	0 (-9 to 9); p =.957	5 (-5 to 15); p =.329	-14 (-22 to -5); p =.003	-12 (-21 to -3); p =.012	-8 (-18 to 1); p =.095	-3 (-13 to 7); p =.559

BL: baseline; Y: years; NS: non-surgery group; S: surgery group.

Appendix 2. Linear mixed models of repeated measures of Knee Injury and Osteoarthritis Outcome Score subscales with time points (baseline, 1-, 3-, 5-, and 10-year follow-up) and intervention group (non-surgery, surgery) treated as fixed effects.

KOOS		Fixed effects	Degrees of freedom		F-statistic	p-value
			Numerator	Denominator		
Intention-to-treat	**Pain**	Time points	4	117.589	54.129	<.001
		Group	1	143.145	0.099	.753
		Time points × Group	4	117.589	7.231	<.001
	**Symptoms**	Time points	4	115.802	42.925	<.001
		Group	1	140.682	0.266	.607
		Time points × Group	4	115.802	4.841	.001
	**Function in daily living**	Time points	4	118.899	33.750	<.001
		Group	1	139.918	0.174	.678
		Time points × Group	4	118.899	3.719	.007
	**Function in sport and recreation**	Time points	4	116.059	38.517	<.001
		Group	1	142.148	0.250	.618
		Time points × Group	4	116.059	2.284	.064
	**Knee related Quality of life**	Time points	4	118.172	58.465	<.001
		Group	1	138.360	0.336	.563
		Time points × Group	4	118.172	2.577	.041
As-treated at 12 months	**Pain**	Time points	4	120.339	51.753	<.001
		Group	1	145.077	0.290	.591
		Time points × Group	4	120.339	6.343	<.001
	**Symptoms**	Time points	4	117.241	40.418	<.001
		Group	1	142.424	0.121	.729
		Time points × Group	4	117.241	4.040	.004
	**Function in daily living**	Time points	4	119.501	31.611	<.001
		Group	1	141.717	0.227	.634
		Time points × Group	4	119.501	5.809	<.001
	**Function in sport and recreation**	Time points	4	117.392	35.991	<.001
		Group	1	143.625	1.155	.284
		Time points × Group	4	117.392	1.833	.127
	**Knee related Quality of life**	Time points	4	120.298	55.346	<.001
		Group	1	139.408	0.031	.861
		Time points × Group	4	120.298	2.818	.028
